# Grape Pomace Extracted Tannin for Green Synthesis of Silver Nanoparticles: Assessment of Their Antidiabetic, Antioxidant Potential and Antimicrobial Activity

**DOI:** 10.3390/polym13244355

**Published:** 2021-12-13

**Authors:** Rijuta Ganesh Saratale, Ganesh Dattatraya Saratale, Somin Ahn, Han-Seung Shin

**Affiliations:** 1 Research Institute of Biotechnology and Medical Converged Science, Dongguk University-Seoul, Ilsandong-gu, Goyang-si 10326, Gyeonggi-do, Korea; rijutaganesh@gmail.com; 2 Department of Food Science and Biotechnology, Dongguk University-Seoul, Ilsandong-gu, Goyang-si 10326, Gyeonggi-do, Korea; gdsaratale@dongguk.edu (G.D.S.); griju22@gmail.com (S.A.)

**Keywords:** grape pomace, silver nanoparticles (AgNPs), in vitro antidiabetic activity, DPPH, antibacterial activity

## Abstract

In nanoscience, the “green” synthesis approach has received great interest as an eco-friendly and sustainable method for the fabrication of a wide array of nanoparticles. The present study accounts for an expeditious technique for the synthesis of silver nanoparticles (AgNPs) utilizing fruit waste grape pomace extracted tannin. Grape pomace tannin (Ta) involved in the reduction and capping of AgNPs and leads to the formation of stable Ta-AgNPs. Various conditions were attempted to optimize the particle size and morphology of Ta-AgNPs which was further analyzed using various analytical tools for different characteristic motives. UV-visible spectroscopy showed a characteristic peak at 420 nm, indicating successful synthesis of AgNPs. Energy disperses spectroscopy (EDS) analysis proved the purity of the produced Ta-AgNPs and manifested a strong signal at −2.98 keV, while Fourier-transform infrared spectrophotometer (FTIR) spectra of the Ta-AgNPs displayed the existence of functional groups of tannin. Zeta potential measurements (−28.48 mV) showed that the Ta-AgNPs have reasonably good stability. High resolution transmission electron microscopy (HR-TEM) analysis confirmed the average dimension of the synthesized NPs was estimated about 15–20 nm. Ta-AgNPs potentials were confirmed by in vitro antidiabetic activity to constrain carbohydrate digesting enzymes, mainly α-amylase and α-glucosidase, with a definite concentration of sample displaying 50% inhibition (IC_50_), which is about 43.94 and 48.5 μg/mL, respectively. Synthesized Ta-AgNPs exhibited significant antioxidant potential with respect to its 2,2′-azino-bis(3-ethylbenzothi-azoline-6-sulfonic acid) (ABTS) (IC_50_ of 40.98 µg/mL) and 2,2-diphenyl-1-picrylhydrazyl (DPPH) (IC_50_ of 53.98 µg/mL) free radical scavenging activities. Ta-AgNPs exhibited extraordinary antibacterial activity against selected pathogenic strains and showed comparable antimicrobial index against ampicillin as a positive control.

## 1. Introduction

Owing to distinctive physical, chemical, optical, catalytic, and magnetic properties, nanomaterials have gained considerable attention for various biological, pharmaceutical, and electronic applications [1]. In recent times, research interest towards nanotechnology has improved which leads to the augmented growth in the production of nanomaterial and its market. Based on their size, nanomaterials are differently grouped, such as nanoparticles, dendrimers, nanotubes and nanofilms [2]. Further, this upsurges the diversity of nanoscale materials. A plethora of advancements in the methodologies for the synthesis of nanoparticles with different characteristics put them together as the most applicable and widely used in materials science. Two conventional production processes, mainly (a) electrochemical and chemical reduction and (b) photochemical and physical vapor condensation, are used for industrial scale nanomaterial production to achieve perfect shapes and higher purity [3]. However, these processes are energy demanding and require hazardous reagents (stabilizing and reducing agents), thus are not eco-friendly and cost-effective. Hence, there is an extensive demand for the definition of less demanding production technologies that would expand the affordability of the whole nanotechnology industry [4,5].

**Figure 1:**
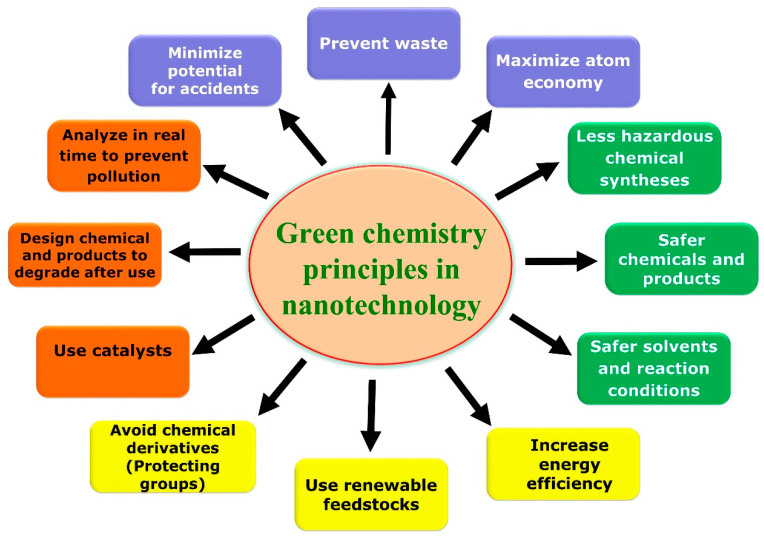
Overview of green chemistry principles applied in green nanotechnology.

Green synthesis is accomplished by combining metal salts with natural reducing agents (such as plant extracts, fruit extracts, and their secondary metabolites), microbial extracts and their by-products (such as vitamins, sugars, and biodegradable polymers) to create nanomaterials [2,6]. The green synthesis of NPs by employing green chemistry principles (Figure 1) is gaining abundant attraction for the development of these future nanosized materials. Plant extract-based nanoparticle synthesis is a non-toxic, eco-friendly, sustainable, and economical way and can perform under aqueous conditions, with low energy requirements and does not require toxic chemicals. Moreover, nanoparticle synthesis by plant extracts is comparatively much faster than the microbial route and easily scalable to produce NPs in huge quantities [7,8]. The fruit and fruit peel extracts contain various pharmacological compounds which function as reducing and capping compounds in the fabrication of different kinds of nanoparticles [9,10,11]. 

Silver nanoparticles (AgNPs) have drawn more attention from various entrepreneurs due to their wide range of scope in numerous industries, such as agriculture, pharmacy, pigments, catalysis, electronics, and cosmetics. Other incredible properties of AgNPs include higher conductivity nature, chemical stability, which increases its potential in pharmaceutical applications mainly, cancer treatment, medical imaging, and drug delivery with reduced undesired toxicity [6,12]. 

Grape (*Vitas vinifera*) can be considered as one of the largest fruit crops; about >67 million tons of grapes are produced per annum globally. Grapes are mainly used for wine production. All through the manufacture of grapes-wine, a major extent of solid organic by-product as a grape pomace is produced (about 40%). Grape pomace signifies a vital source of phenolic antioxidants and can be utilized as an animal feed supplement with health promoting factors [13,14]. However, utilization of the huge amount of grape pomace is still scarce, thus it accumulates near wine industries as a waste product and causes environmental and disposal complications. High levels of condensed tannins are held back as residue, based on the low extraction during winemaking. Tannin is a polyphenolic compound, having human health benefits due to higher antioxidant potential [11,15]. The present study was intended for silver nanoparticles green synthesis using *Vitis vinifera* (extracted grape pomace tannin), which has not been studied well in nano research. Further, the influence of several operational factors in the synthesis of Ta-AgNPs were critically examined and optimized. Characterization of Ta-AgNPs with regard to the size and size distribution morphology and structure of particle size was accomplished using various standard analytical techniques. Ta-AgNPs were assessed for multi biogenic potentials in terms of in vitro antidiabetic and antioxidant activities by employing standard enzyme assays. Finally, the antimicrobial efficacy was investigated against pathogenic bacteria cultures to raise their potential applications in biomedical sectors.

## 2. Materials and Methods

### 2.1. Grape Tannin Extraction and Reagents

Grape pomace (GP) was procured from the local wine industry, washed and oven dried, until a persistent weight was attained. Further, the dried GP was cut into small pieces and finely ground. Tannin extraction from grape pomace was performed using the methodology reported elsewhere [16]. The finely ground grape pomace was added in water comprising of Na_2_CO_3_ (2.5%) and Na_2_SO_3_ (2.5%) aqueous base solution at 80 °C for 4 h followed by the filtration, and the resultant portion was spray-dehydrated and the resulting powder was utilized for the synthesis of silver nanoparticles and for subsequent procedures. Silver nitrate (AgNO_3_), ascorbic acid, 2,2-diphenyl-1-picryhydrazyl (DPPH), 2,2′-Azino-bis(3-ethylbenzothiazoline)-6 sulfonic acid (ABTS), sodium potassium tartrate, 3,5-dinitrosalicylic acid (DNS), acarbose, α-glucosidase, and α-amylase, were procured from Sigma-Aldrich, St. Louis, MO, USA. All other reagents and chemicals used for the study were of analytical grade quality and of higher pureness. Double distilled water was used throughout in all the experiments for solution preparations (Millipore Corporate, Billerica, MA, USA).

### 2.2. Green Mode Synthesis of Ta-AgNPs, Optimization of Conditions and Stability Studies

The synthesis of Ta-AgNPs was performed in the aqueous grape pomace extracted tannin, which is a reducing agent and silver nitrate (AgNO_3_), as the precursor compound. Grape tannin (1000 ppm) and the AgNO_3_ solution (1 mM) were individually prepared. Appropriate volumes of tannin and AgNO_3_ solution (ratio of 1:10) in a flask were gradually mixed at 30 °C on a magnetic stirrer. At regular time intervals, the samples were collected from the reaction mixture and studied for their absorption spectrum by employing UV-visible absorption spectroscopy. The change in color (light brown and became darker) was also noted. In order to improve the properties and analytical merit of Ta-AgNPs, the reaction conditions, such as pH value (2, 3, 4, 5, 6, 7, and 8), reaction time (0, 5, 10, 20 and 30 min), concentration of AgNO_3_ (0.5, 1.0, 2.0, and 2.5 mM), and tannin concentration by means of varied mixing ratios of Ta:AgNO_3_ (1:1, 1:5, 1:8, 1:10, 1:15, and 1:20), were inspected in a detailed manner. The optimization of NP synthesis factors was diversified one at a time by upholding the other variable stable factors. Under optimized conditions the produced Ta-AgNPs were concentrated and separated from the reaction mixture by setting a centrifuge at 12,000 rpm for 20 min (Labogene, 1736R, Lillerød, Denmark). The resulting Ta-AgNPs pellet was washed with distilled water to exclude the impurities and further dehydrated in an oven (60 °C) for analytical studies and biogenic potentials. The synthesized Ta-AgNPs were observed for up to 3 months for their stability, by keeping them at room temperature conditions and by applying the procedure reported earlier [9]. All experiments were conducted in triplicate sets.

### 2.3. Characterization of Ta-AgNPs

The optical property of Ta-AgNPs was determined between 300 nm and 700 nm at regular time intervals by using a UV-visible spectrophotometer (Optizen, Model-2120, Daejeon, Korea). The participation of several functional groups of extracted tannin during the bio-reduction and synthesis of Ta-AgNPs was analyzed using a Fourier-transform infrared spectrophotometer (Perkin-Elmer, Norwalk, CT, USA). Meanwhile, the spectrum of energy disperses spectroscopy (EDS; JEOL-64000, Tokyo, Japan) and element distribution of Ta-AgNPs was also measured. Zeta potential of Ta-AgNPs was assessed by a zeta potential analyzer (ELS-8000, Tokyo, Japan). A high resolution transmission electron microscopy (HR-TEM, Tecnai G2 20 S-TWIN, FEI Company, Loughborough, UK) was used to analyze the size, shape, and exterior morphology characteristics of Ta-AgNPs. The particle size of Ta-AgNPs was measured using the standard procedure [9].

### 2.4. In Vitro Antidiabetic Potential of Synthesized Ta-AgNPs

Antidiabetic potential of synthesized Ta-AgNPs was evaluated by measuring the inhibition capability against two types of carbohydrate hydrolyzing enzymes (α-amylase and α-glucosidase). For the α-amylase enzyme assay, a diverse quantity of synthesized Ta-AgNPs (20, 40, 60, 80, and 100 µg/mL; about 1 mL) was added to 1 mL starch solution and kept at room temperature (30 °C) for 10 min. Through adding 1 mL of dinitrosalicylic acid color reagent, the reaction was stopped and then kept in a boiling water bath for 10 min and further cooled. Finally, the absorbance was checked for the mixture at 540 nm in a colorimeter. The α-glucosidase assay was performed according to the standard procedure and determined the inhibition of the enzyme activity by the Ta-AgNPs [17]. For the determination of both the enzyme assays, acarbose was considered as standard. Three replicated determinations were performed, and the averaged results were recognized to determine the antidiabetic potential of Ta-AgNPs. The enzyme activity was specified as of IC_50_ value (articulated as the definite concentration of a sample displaying 50% inhibition).

Free radical scavenging (%) = [(AC − AT)/AC] × 100

AC = absorbance of control; AT = absorbance after exposure to Ta-AgNPs.

### 2.5. In Vitro Antioxidant Potential of Synthesized Ta-AgNPs

Antioxidant potentials of ascorbic acid (as standard), extracted tannin, and synthesized Ta-AgNPs were investigated by quantifying the free radical scavenging activity against 2,2-diphenyl-1-picrylhydrazyl (DPPH) and 2,2-Azino-bis(3-ethylbenzothiazoline)-6 sulfonic acid (ABTS). The scavenging activity enzyme assays were conducted by employing the formerly described standard protocol [18]. The antioxidant activity of all samples was measured by taking the average mean values and standard deviation values and their scavenging potential was specified as of IC_50_ value using the previously described procedures.

### 2.6. Antimicrobial Activity

In vitro estimation for antibacterial efficacy of bio fabricated Ta-AgNPs was performed using Gram-negative and Gram-positive bacteria cultures (*Escherichia coli* and *Staphylococcus aureus*) through the standard Kirby–Bauer disc diffusion procedure [19]. First, in the nutrient broth the cultures were revived at 37 °C overnight to attain optimum O.D. (0.4) at 600 nm. Freshly grown overnight cultures were inoculated (100 μL) and swabbed using sterilized cotton swabs on agar plates. Further, using sterile filter paper discs, extracted tannin, Ta-AgNPs, and Ampicillin were kept on the inoculated agar medium. For the uniform perfusion of the samples initially, the petri plates were left to stand for 1 h, then incubated overnight at 37 °C for 24 h for bacterial culture growth. Zone of inhibition was calculated (mm) using a uniform scale round the disc infused with extracted tannin, Ta-AgNPs, and Ampicillin. Extracted tannin aqueous form was reflected as the negative control and ampicillin was applied as a positive control. The antimicrobial index of Ta-AgNPs against each pathogenic bacteria was measured and interpreted using the mentioned formula [20].

Antimicrobial index = (inhibition zone by Ta-AgNPs/inhibition zone by ampicillin) × 100

### 2.7. Statistical Analysis

All the experiments were conducted in three sets and the data of all the results calculated values were deliberated as mean ± standard error mean (SEM). The data obtained were inferred by using the one-way analysis of a variance (ANOVA) test convoyed by a Tukey–Kramer multiple comparisons test.

## 3. Results and Discussion

### 3.1. Ta-AgNPs Synthesis 

**Figure 2:**
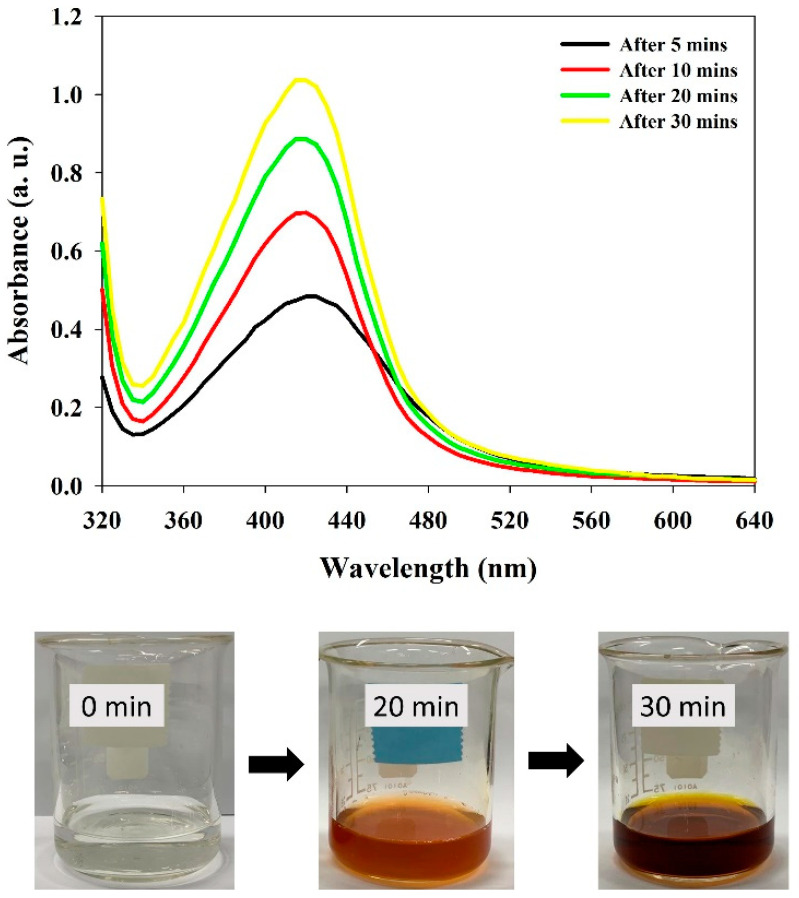
UV-visible absorption spectrum of synthesized Ta-AgNPs and the color changes in the reaction mixture at different time intervals.

**Figure 3:**
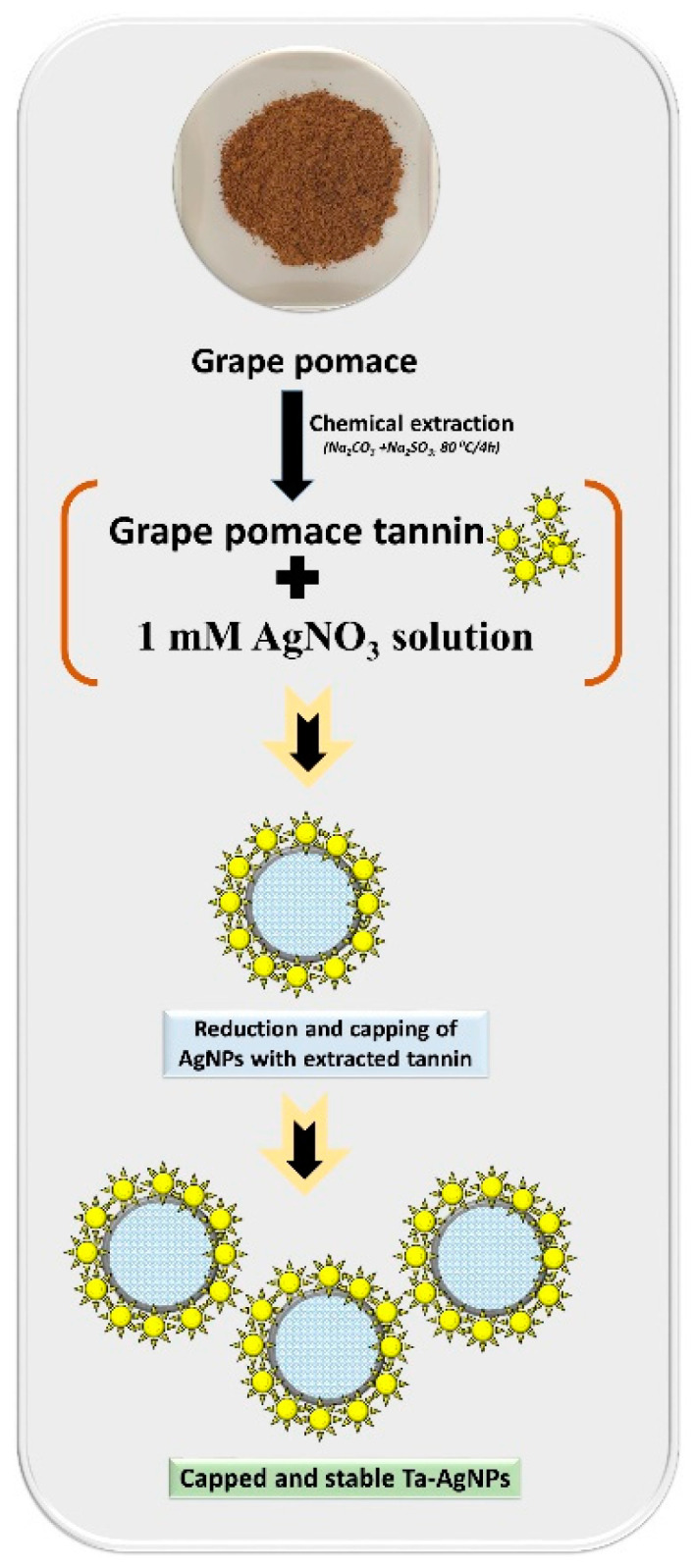
Schematic representation of the grape pomace tannin mediated synthesis of Ta-AgNPs.

Tannin as a naturally occurring predominant phytomolecule in grape pomace possesses higher antioxidant activity [13]. Tannins were extracted from grape pomace and utilized for the synthesis and fabrication of Ta-AgNPs. Synthesis of Ta-AgNPs was visually marked by checking the color alteration of the reaction solution, from slight brown to dark brownish red, and also subjected to surface plasmon resonance (SPR) analysis by using UV-visible spectroscopy at various time intervals. The spectral analysis manifested a distinct SPR peak at 420 nm after 30 min of incubation (Figure 2). The absorption spectral peaks in the range of 410–450 were used for the characterization of the Ag nanoparticles [21]. In line with the Mie theory, spherical nanoparticles show only a single surface plasmon resonance (SPR) band, which supports our results [12]. The phenolic compounds are mainly responsible for this chelating ability due to the nucleophilic nature of their aromatic rings. During the NP synthesis process, Ag^+^ ions are captured and chelated by extracted tannins, which subsequently undergo a reduction, nucleation, and capping process, resulting in the development of stable Ta-AgNPs [22]. The detailed schematic representation of the grape pomace tannin mediated synthesis of Ta-AgNPs has been presented in Figure 3.

### 3.2. Optimization of Ta-AgNPs Synthesis Process Parameters

**Figure 4:**
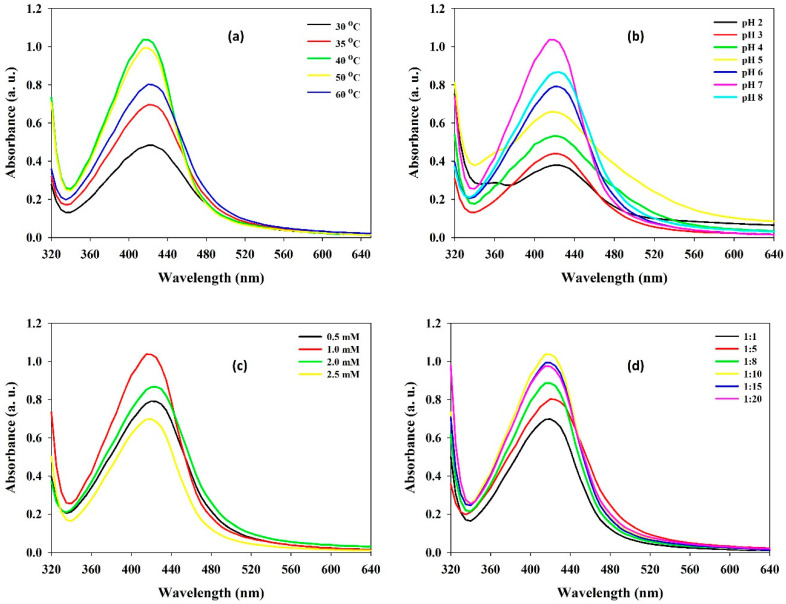
The effect of various influencing operational parameters (**a**) incubation temperature; (**b**) initial pH; (**c**) AgNO_3_ concentration; and (**d**) Ta:AgNO_3_ ratio on the synthesis of Ta-AgNPs and their UV-visible spectra.

To regulate the size and morphology of nanoparticles, the process parameters can be either optimized or modified. The solution pH, temperature, reaction time, and phytochemical quantity are vital factors affecting the size, shape, and efficiency of the NP synthesis process [22,23]. Temperature is another significant process parameter, with an increase in temperature, the development of nucleation centers increases, which eventually upsurges the rate of nanoparticle synthesis. In this study, the SPR peak of produced Ta-AgNPs showed an increase in the absorption intensity from 30 to 40 °C, while no difference at 40 °C and 50 °C was noted (Figure 4a). At a higher temperature (60 °C) a sharp reduction in SPR peak was discerned. Similarly, in other studies of AgNPs synthesis using plant extract, low reaction temperatures for stable nanoparticles synthesis relative to high temperatures were required [24].

For instance, alteration in the pH leads to change in the overall charge of bioactive phytomolecules, which in turn facilitates their binding affinity and hence bioreduction of metal ions into nanoparticles. During the synthesis of Ta-AgNPs at different pH of the reaction media, maximum SPR distinct peak was recorded at pH 7.0 while other pH values found were not significant in the synthesis of Ta-AgNPs (Figure 4b). Nindawat and Agrawal [25] showed that *Arnebia hispidissima* extract promoted synthesis of small size AgNPs at alkaline pH levels of 7.0, 9.0 and 11.0, whereas flat UV spectrum was observed at pH 3.0, which support our results. The effect of the initial concentrations of silver nitrate from 0.5 mM to 2.5 mM on Ta-AgNPs synthesis was studied. The results suggested a distinct upsurge peak up to 1.0 mM (Figure 4c). However, as the increase in silver nitrate concentration accelerates to decrease the SPR value, it might be due to the agglomeration of synthesized NPs.

High concentrations of polyphenols avoid coalescence and aggregation of nanoparticles; thus, determination of proper tannin concentration is essential. The effect of varying concentrations of tannins on Ta-AgNPs synthesis was investigated by making different mixing ratios of tannin and AgNO_3_. The results suggest that lower concentrations of tannin were found to be effective and the maximum SPR was noticed at a tannin and AgNO_3_ 1:10 ratio (Figure 4d). However, further decreasing the tannin concentration was found to be not effective in Ta-AgNPs synthesis [26]. The obtained results can be considered as noteworthy, which can help make the process worthwhile and commercially applicable. In accordance with the comprehensive results, the optimized parameters for Ta-AgNPs synthesis were 40 °C temperature, pH: 7.0, 1.0 mM AgNO_3,_ Tannin/AgNO_3_ ratio: 1:10, and 30 min incubation time, and were selected for further experimentation.

#### 3.2.1. Analytical Studies of Synthesized Ta-AgNPs

Generally, nanoparticles are characterized on the basis of their morphology, size, surface area, zeta potential and dispersity index. A homogenous and monodispersed solution of these nanoparticles is extremely important for numerous applications. 

**Figure 5:**
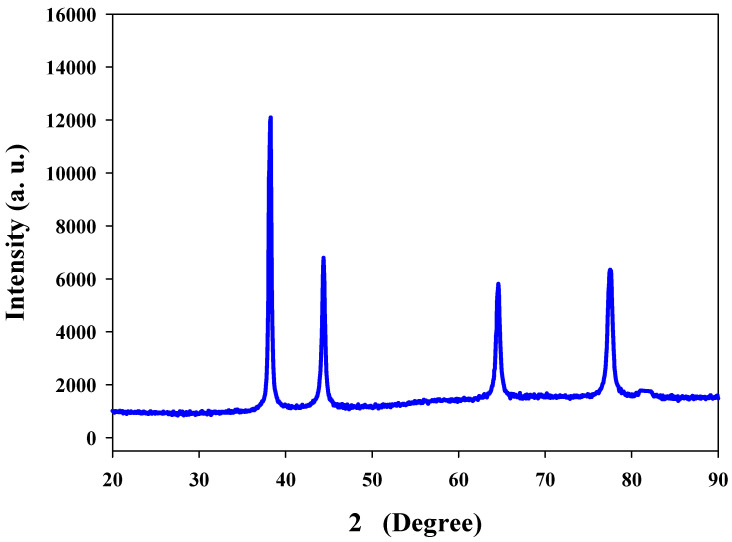
XRD pattern of Ta-AgNPs synthesized under optimized conditions.

XRD is generally advantageous to analyze the purity and monocrystalline nature of the nanoparticles [9,27]. X-rays penetrate deep into nanoparticles that generate a diffraction pattern, which is further compared with the standard for the collection of structural details. Measurements for the XRD of the Ta-AgNPs showed four distinct peaks at 2θ angles of 38.12, 46.15, 64.75, and 76.54 attributes to (111), (200), (220), and (311) (Figure 5). The XRD spectrum of the synthesized AgNPs showed 2θ peak corresponding to 111 (at 38.1°) Bragg reflections of silver and also confirms the presence of face centered cubic (FCC) crystal structure. The results are in accordance with the silver nanoparticle synthesized by leaves of *Panax ginseng* confirmed the crystalline nature with FCC structure [28]. 

#### 3.2.2. FTIR Analysis 

**Figure 6:**
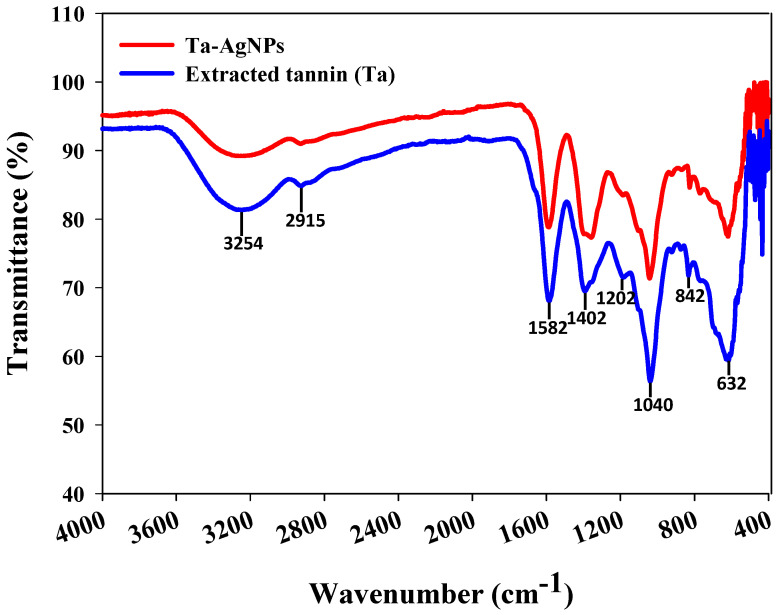
FTIR spectra of extracted tannin and Ta-AgNPs synthesized under optimized conditions.

FTIR spectroscopy is used for the portrayal of the surface chemistry of nanoparticles and to identify the active functional groups [18]. FTIR analysis was carried out to discover the functional group deviations amongst grape pomace extracted tannins before and after it is fabricated on the surface of the AgNPs. FTIR spectra of extracted tannin and Ta-AgNPs are represented in Figure 6. The presence of broad absorption bands around 3254 cm^−1^ in both extracted tannin and synthesized Ta-AgNPs corresponds to the hydroxyl group (O-H) of polyphenol constituent [29]. Two peaks at 1582 and 1402 cm^−1^ are representative of the aromatic ring structures, whereas a small peak at 2922 cm^−1^ relates to the stretching of C-H [11]. Moreover, the peaks in the region 1000 to 1300 cm^−1^ exhibited for aromatic ring vibration [16]. In a study, analogous outcomes have been observed in the silver nanoparticle synthesis using different forms of tannins (condensed and hydrolysable) extracted from chestnut, mangrove and quebracho [30]. FTIR results suggest the presence of polyphenolic and aromatic constituents of extracted tannins on the surface of Ta-AgNPs, which are involved in the reduction, capping and stabilizing the synthesized Ta-AgNPs.

#### 3.2.3. EDS and Zeta Potential Analysis 

**Figure 7:**
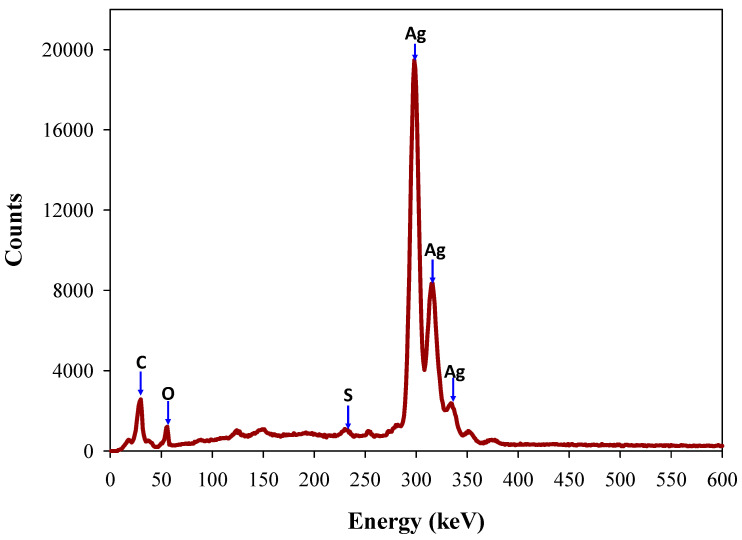
Energy dispersive spectroscopy (EDS) analysis of Ta-AgNPs synthesized under optimized conditions.

Energy dispersive spectroscopy (EDS) is commonly used to calculate elemental composition and the purity investigation of synthesized AgNPs. The EDS measurement of the synthesized Ta-AgNPs showed the strongest absorption peak at 2.98 keV corresponds to metallic silver due to surface plasmon resonance by silver atoms (Figure 7). The other minor peaks of C and S are related to tannin molecules, suggesting its involvement in the synthesis and fabrication of Ta-AgNPs. Few other studies confirmed that the adsorption of silver has been observed around 3 keV, which corresponds to the binding energy of elemental silver [11,28,31].

Further, the synthesized Ta-AgNps were studied by using zeta potential for the determination of surface charge. The analysis results showed the presence of higher negative surface charge (−28.48 mV), which can prevent the NPs from agglomerating. Some researchers suggest that nanoparticle zeta potential values > 30 or <−30 are comprised of high levels of stability [32]. This property is useful for the stability of synthesized NPs due to which SPR spectrum of Ta-AgNPs remain stable for about 3 months, and thus can be used in a continued way. Analogous zeta potential value −28.4 mV was perceived in the silver nanoparticles synthesized using *Vitis vinifera* skin extract [11,30].

#### 3.2.4. HR-TEM Analysis

**Figure 8:**
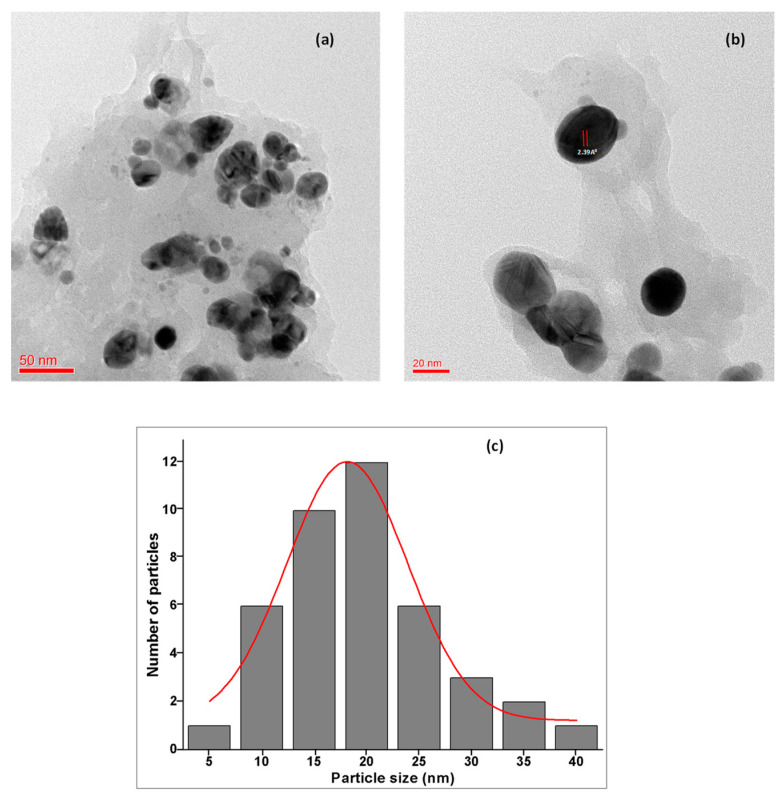
HR-TEM images of Ta-AgNPs: (**a**) at 50 nm; (**b**) at 20 nm amplification; and (**c**) average particle size histogram of the Ta-AgNPs produced under optimized conditions.

Electron microscopy is another universally used technique for the analysis of the size and morphological characterization of nanoparticles. The TEM micrographs at different 50 nm and 20 nm magnifications revealed that the synthesized Ta-AgNPs are spherical in shape and uniformly distributed in the sample (Figure 8a,b), which aligns well with XRD and UV-visible spectroscopy results. TEM images also showed the presence of dark caps on the outer layer of nanoparticles which was due to the occurrence of tannin biomolecules on the surface of synthesized Ta-AgNPs. The particle histogram suggested the maximum NP size is in the range of 15 to 20 nm (Figure 8c), which increases its potential applicability in various sectors. Similar types of observations were recorded in the silver nanoparticles synthesized using *Acacia nilotica* leaf extract, jasmine flower extract, and aqueous extract of *Dracocephalum kotschyi* Boiss, respectively [31,33,34,35].

### 3.3. Biogenic Potential of Synthesized Ta-AgNPs

The biogenic potential of the green synthesized Ta-AgNPs was assessed by investigating their antidiabetic, antioxidant, and antibacterial activities.

#### 3.3.1. Antidiabetic Potential 

**Figure 9:**
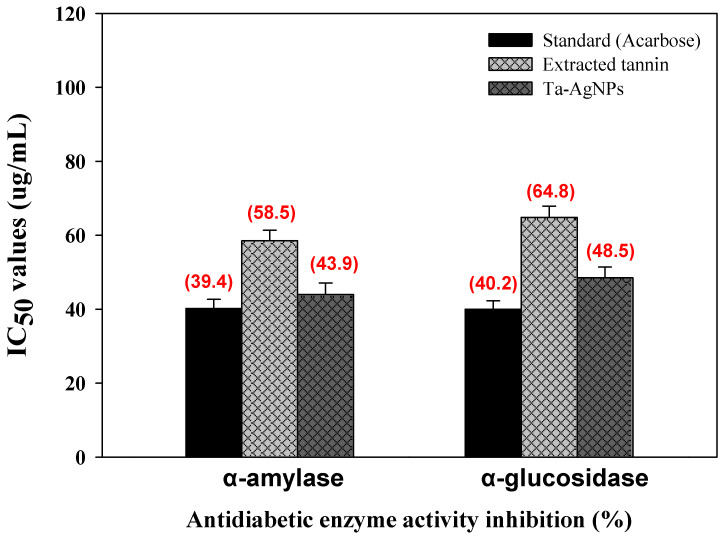
Antidiabetic potential of synthesized Ta-AgNPs, extracted tannin, and standard (acarbose) against α-amylase and α-glucosidase and their IC_50_ values.

The green synthesized silver nanoparticles have been established as highly stable and useful candidates for drug carriage because of their ultra-small size and unique physicochemical properties [7]. The capability to fine-tune the surface charge of the nanoparticle helps in targeting specific locations and the controlled release of drugs. α-amylase and α-glucosidase are accountable for hydrolyzing oligosaccharides or polysaccharides into α-d-glucose which are adsorbed by intestinal cells, leading to postprandial hyperglycemia [36]. This unusual higher sugar level is responsible for the occurrence of type 2 diabetes (T2DM) which is troublesome and not easy to control. Acarbose, voglibose, and miglitol found clinically effective drugs to restrain or to treat T2DM by inhibiting the carbohydrate degrading enzymes [37]. However, these drugs are costly and also show adverse effects. To overcome this, there is a crucial requirement to establish effective NPs coated with natural products to control T2DM and sequential disorders. The synthesized Ta-AgNPs showed effective inhibition for both α-amylase and α-glucosidase enzyme activities in a dose dependent mode. The half-inhibitory concentration (IC_50_) of Ta-AgNPs, extracted tannin and standard drug acarbose were determined and shown in Figure 9. The IC_50_ value of Ta-AgNPs and acarbose for α-amylase and for α-glucosidase were 43.94 and 40.2 μg/mL and 48.5 and 40.0 μg/mL, respectively (Figure 9). *Holoptelea integrifolia* leaves mediated AgNPs showed antidiabetic potential against α-amylase with significant 86.66% inhibition in enzyme activity [38]. In this study, the authors have proposed that synthesized AgNPs inhibit ATP-sensitive K^+^ channel mechanism in beta cells of the pancreas. 

#### 3.3.2. Antioxidant Potential 

**Figure 10:**
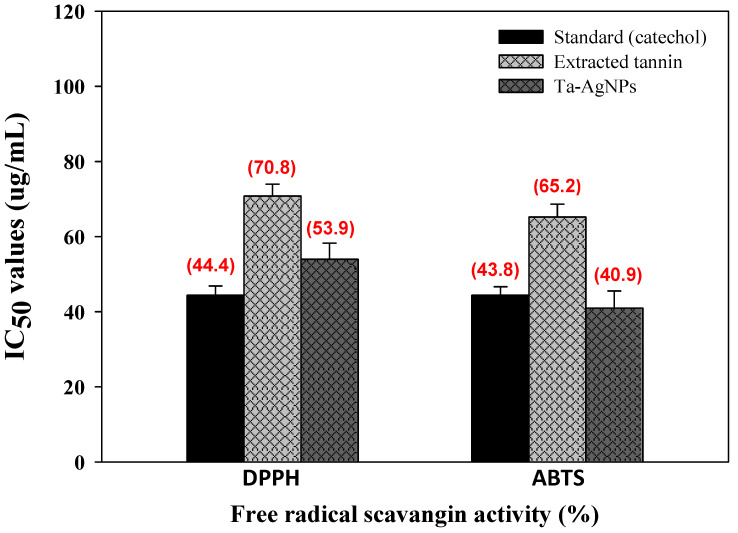
Antioxidant potential in terms of scavenging activity of synthesized Ta-AgNPs, extracted tannin, standard (catechol) against highly stable DPPH and ABTS and their IC_50_ values.

Free radical generation is responsible for the existence of several pathological diseases, for instance cancer, heart disease, diabetes, Alzheimer’s, hypertension, atherosclerosis, and aging [8]. The bio-synthesized Ta-AgNPs demonstrated significant antioxidant perspectives in terms of radical scavenging activities against stable free radical DPPH and ABTS and are presented in Figure 9. Ta-AgNPs displayed promising DPPH and ABTS free radical-scavenging activities in a concentration dependent mode. The standard catechol and synthesized Ta-AgNPs for DPPH and ABTS showed IC_50_ values (44.4 and 43.8 µg/mL) and (53.9 and 40.9 µg/mL), respectively (Figure 10). In both enzymes only extracted tannin was found less effective and documented higher IC_50_ value (70.8 and 65.2 μg/mL) relative to standard and Ta-AgNPs (Figure 10). In the case of grape seed and apple tannins, grape seed tannins displayed significantly more antioxidant activity than apple tannins [39]. The free-radical scavenging potential of biosynthesized nanoparticles and their application for the cure of different pathological conditions have been studied in vitro by several researchers [9,25,40]. The significant antidiabetic and antioxidant potential of Ta-AgNPs due to the tannin molecules are involved during the synthesis and fabrication of NPs. These molecules enhance the surface area of NPs, and their proper interaction leads to significant antidiabetic and antioxidant activities.

#### 3.3.3. Antibacterial Potential 

There has been emerging attention paid to exploring substitute approaches to developing novel antimicrobial agents since there is a continuous rise in multidrug resistant bacteria due to excess antibiotics use, mutation, and environmental circumstances [6,41]. The smaller sized nanoparticles have a superior binding surface area relative to larger NPs and show more potent antimicrobial activity. AgNPs found a potent antimicrobial agent. The synthesized Ta-AgNPs exhibited potential antibacterial activity towards the designated strains. The results are expressed as zone of the inhibition (ZOI) to define the comparative antibacterial potential of Ta-AgNPs and the results have been presented in Table 1. The obtained results revealed that the synthesized Ta-AgNPs executing significant antibacterial activity and can be used in the development of antibacterial drugs. It was supposed that Ta-AgNPs exhibited significant antibacterial effect, owing to its ability to penetrate the membrane and interact with cellular components, mainly destruction of respiratory enzymes, destruction of electron transport process, and DNA function, which leads to growth inhibition. Still, additional research is essential to understand the exact mechanisms of antibacterial activity by NPs. Disruption of membrane potential leads to cytoplasmic leakage, which results in the release of proteins and lipopolysaccharide molecules, and finally lysis of bacterial cells was observed [42]. AgNPs synthesized using the leaf extract of *Neurada procumbens* showed noteworthy antimicrobial activity against multidrug resistant Gram-negative pathogens *Klebsiella* *pneumoniae, Acinetobacter baumannii* and *Escherichia coli* [43]. Moreover, Hashim et al. [11], Escárcega-González et al. [41], and Kim et al. [44] also conveyed antibacterial activity of plant extract mediated AgNPs against *S. epidermidis*, *S. aureus*, *Listeria monocytogenes*; *E. coli*, *P. aeruginosa*, *P. aeruginosa, B. subtilis*; and *E. coli*, and *S. aureus*, respectively. 

## 4. Conclusions

The fruit waste grape pomace extracted tannin was exploited for the synthesis of Ta-AgNPs for the potent approach as more cost effective and non-toxic, as well as useful in lessening the burden of grape pomace waste. Optimization of synthesis parameters and their characterization using various standard analytical techniques was performed. The results suggest the synthesized NPs are spherical, monodispersed and highly stable. Tannin fabricated AgNPs showed significant antidiabetic potential by inhibiting the marker carbohydrate hydrolyzing enzymes, namely α-amylase and α-glucosidase. Additionally, Ta-AgNPs showed promising antioxidant potential and antibacterial activity against pathogenic microorganisms. In consonance with all-inclusive results, Ta-AgNPs displayed a wide array of biological solicitations and can be recognized as attractive, eco-friendly material for its possible use in drug delivery, diabetes treatment, antibacterial activity, and cancer therapy, without negative effects.

## Figures and Tables

**Table 1:** Assessment of antimicrobial activity of Ta-AgNPs against pathogenic microorganisms.

	Zone of Inhibition (mm)			
Pathogen	Ta-AgNPs concentration (20 μg/mL)	Positive control (20 μg/mL)	Negative control (20 μg/mL)	Antimicrobial index (%)
*Escherichia coli*	14.2 ± 0.48	15.2 ± 0.52	4.85 ± 0.98	93.4 ± 2.05
*Staphylococcus aureus*	11.1 ± 0.82	13.7 ± 0.65	4.15 ± 0.68	81.0 ± 2.35

Positive control—Ampicillin; negative control—extracted tannin. NA—no activity. Values are mean ± standard error of three replicates.
